# Lucid dreams: their advantage and disadvantage in the frame of search activity concept

**DOI:** 10.3389/fpsyg.2015.01472

**Published:** 2015-09-30

**Authors:** Vadim S. Rotenberg

**Affiliations:** Formerly affiliated with Abarbanel Mental Health Center, Tel-Aviv UniversityBat-Yam, Israel

**Keywords:** dream function, lucid dreams, REM sleep, search activity, helplessness

## Abstract

Search activity (SA) is the behavioral and mental activity that is oriented to changes of the environment or of the subject's view and approach to the environment according to personal needs without the definite probability forecast of the outcomes of such activity, but with a regular consideration of the outcomes in the process of active behavior. Dream's lucidity (the subject's realization that he/she is dreaming) protects dreamer from awakenings during emotionally disturbing or frustrating dreams, because lucid dreams allow subject to feel separated from the dream events that may cause a feeling of helplessness. Due to such a protection from awakenings that can bring subject back to the frustration in wakefulness, subject can turn in the further sleep to normal non-lucid dreams that are restoring subject's SA in the subsequent wakefulness (activity in the uncertain situation with the feedback between behavior and its outcome). It is the advantage of lucid dreams. Their disadvantage is that due to the separation from the dream events that are in lucid dreams accepted as rationalized dreams, not as real stories where the dreamer acts like in wakefulness, their ability to restore SA is decreased until they are not displaced by the normal non-lucid dreams accepted as real stories.

## Introduction: the restoration of search activity in dreams

The problem of dream lucidity is discussed in many scientific publications during the recent few decades. In this article I am not going to cover all aspects of this topic. I would only like to consider the functional role of this relatively rare phenomenon (in comparison to non-lucid dreams) in the context of the general function prescribed to rapid eye movement (REM) sleep and dreams by the Search Activity Concept (SAC, Rotenberg and Arshavsky, 1979; Rotenberg, 2009, 2013).

By search activity (SA) we understand behavioral and mental activity that is oriented to changes of the environment or of the subject's view and approach to the environment according to personal needs without the definite probability forecast of the outcomes of such activity, but with a regular consideration of the outcomes in the process of active behavior. In humans and in other animals with a high level of mental activity complexity, SA is a very important part of subject's relationships with the complicated and dynamic world, and determines the adaptation to it including an overcome of numerous stressful events. Such adaptation cannot be achieved in the process of stereotyped behavior that is relevant only in conditions, where the definite forecast of the outcome of behavior is available. SA is a basis of the personal development and of the development and evolution of the society. It requires a lot of energy, but this energy is restored through the positive feedback between search activity and brain biochemistry (see Rotenberg, 2009).

Our investigations performed on animals (Rotenberg and Arshavsky, 1979) have shown that behavior that includes SA increases the resistance to different forms of artificially induced somatic disorders. On the other hand, renunciation of search that displays itself in giving up and helplessness in front of stressful events and obstacles that subject is unable to cope with, decreases body resistance and causes numerous somatic disorders, typically estimated in humans as psychosomatic diseases, such as peptic ulcer and essential blood hypertension (see Rotenberg, 2009). In humans, renunciation of search is also typical for depression (Rotenberg and Cholostoy, 2004; Rotenberg et al., 2007, 2008).

In real life, subjects are often meeting distressing situations and conditions that they are unable to change or obstacles that prevent achievements of their personally important goals, and such conditions can cause renunciation of search. Obviously, if SA is so important for the survival and development, there must be a natural special biological mechanism that restores the adaptive abilities to cope with numerous stressful events in such conditions. Without this mechanism, any occasional stressful situations even restricted in time, or any terminal but unavoidable obstacle would have a fatal outcome for the subject. According to the SAC (Rotenberg and Arshavsky, 1979; Rotenberg, 2009, 2013), REM sleep dreams in humans and in other animals with a high level of mental activity complexity are performing such restorative function. For instance, healthy subject can go to sleep in a state of giving up with a feeling of being unable to continue search for solution of a given problem and to overcome obstacles, and although after awakening the problem by itself is still not solved, subject is ready to continue efforts to overcome it—in a contrast to the state just before sleep. In our investigations, we have found that in such cases, REM sleep latency is usually decreased, REM sleep percentage in the night sleep is increased, and eye movement density in REM sleep is increased, and when healthy subjects awoke in REM sleep, they often present reports about vivid dreams where they have performed an intense virtual activity (see Rotenberg, 2009).

During SA, the process of this activity by itself is more important than its pragmatic outcomes. The main task of this process is not to stop, not to give up in front of episodic failures. For this reason, REM sleep dreams represent the most relevant condition for the restoration of SA. In dream, subject is isolated from the outward external world including those stressful events that already caused in the previous wakefulness renunciation of search after many failures. SA in dream is a virtual activity, based on imagination. It means that it can be very flexible, polymorphic and chaotic; subject can jump in his dreams from one imagined situation to another and even pass many failures in the process of this activity, because being only virtual, but not real, these failures in reality have no dangerous outcomes for the subject—the only task is not to stop the process. The direction of search can be changed at every moment. The decision to change the direction of search is not made on the basis of rational analysis—it is a sudden impulsive decision, and it costs nothing for the dreamer. This search may be in some way, directly or symbolically, related to the problems that dreamer met in the previous wakefulness. For instance, it may reflect the discharge of suppressed (repressed) motives, otherwise unacceptable for the waking consciousness (according to the psychoanalytical approach). It was confirmed in some investigations. For example, Grieser et al. (1972) have shown that REM sleep deprivation in healthy subjects increases repression and causes emotional tension (anxiety). But often dreams has nothing to do with repressed motives, and are oriented on the occasional imagined goals.

From my point of view, the repression of unacceptable motives is a form of human's renunciation of search—of search for the ability to discharge these motives in real behavior, to realize them or to integrate them into socially acceptable motives, in order to protect the holistic behavior and the Self-concept. However, for the virtual SA in dreams, the topic of the renunciation of search in previous wakefulness is less important than the renunciation by itself, and even if repression of unacceptable motives represents the renunciation of search, the compensatory search in dreams may deal with conditions that are not related to repressed motives. Moreover, the task of the dream is often to turn subject away from the topic that brought him/her to the feeling of helplessness.

REM sleep and dreams characterizes not only humans with their inner motivational conflicts, but also other animals with a high level of mental activity complexity. If we are looking for a common function of REM sleep and dreams in both humans and other animals with a high level of mental activity complexity, restoration of SA seems to be a relevant explanation. In REM sleep, subject is separated from the external reality, from obstacles, mistakes, and punishments that caused renunciation of search, and this separation helps SA in dreams to start as from the very beginning (Rotenberg, 2009; Hobson et al., 2014). However, sometimes the state of giving up and a feeling of helplessness caused by the unavoidable problems in wakefulness are both so strong, so that it spreads on the virtual reality of dreams (Hobson et al., 2014). Painful failures in dream stories can in such conditions cause the incorporation of renunciation of search into these stories. It is common in patients with mental and psychosomatic disorders, but it can appear also in dreams of normal subjects that for occasion, sometimes include in the process of chaotic search some virtual conditions that subject is unable to overcome just now. In this condition, dream is not only accompanied by negative emotions like fear, anger and hate that are often presented in normal dreams as parts of dream stories and stimulate active virtual search, but became occupied by the feeling of inability to cope with these problems that are for dreamer in the non-lucid dream not virtual but real (Rotenberg, 1988, 2009; Goldstein and Walker, 2014). In such a situation, the dreamer is sometimes unable to jump over the traumatic dream images that cause a feeling of being a victim, and to turn to another images, and this feeling suppresses the SA in the dream. The ability to create new imaginative stories in dreams that can restore SA may be restricted also due to the low right hemisphere functional activity (Rotenberg, 1995). The behavior in dream in such cases becomes by itself frustrating and increases feeling of helplessness instead of overcoming it. Dreams turn into nightmares that are leading to awakenings, dreamer awoke with a strong feeling of helplessness and instead of being compensated this state becomes elevated in dream (Goldstein and Walker, 2014). Thus, it may be a vicious circle between helplessness in wakefulness and in dream.

In some cases, dreams are losing their vividness, their content became poor and restricted, and game with images disappeared. It represents the functional inefficiency of dreams, and is leading to a further degradation of dreams and to a progressive disappearance of dream content. It is exactly what we have found in patients with different mental disorders (depression, neurotic anxiety, etc.) and with psychosomatic disorders, and we come to the conclusion that it is a sign of the REM sleep functional insufficiency, exhaustion of dreams that plays an important role in the pathogenesis of these diseases (see Rotenberg, 2009). Like in wakefulness, renunciation of search in dreams is working with a positive feedback and negative outcome, meaning that it progressively increases itself.

## The essence of lucid dreams and their disadvantage

After this large introduction let us turn to the main topic of the presentation, to the advantage and disadvantage of the so-called “lucid dreams.” These are dreams, in which dreamer, in opposite to the more common and typical non-lucid dreams realizes on the conscious level that he/she is dreaming and participating in the own virtual reality, as if looking on him-/herself as a dreamer from outside. This phenomenon has a neurophysiological basis. In opposite to the non-lucid dreams, that are characterized by a decreased activity of the dorsolateral prefrontal cortex (Maquet et al., 2005) responsible for the self-control and for the formation of the Self-Concept, lucid dreams are characterized by the relative increase of the functional and physiological activity of this brain structure, and are according to this activity similar to wakefulness (Kahn and Hobson, 2005; Hobson, 2009; Voss et al., 2009).

However, it does not mean that consciousness is totally absent during non-lucid dreams. In non-lucid dreams, subject is aware while dreaming about all events in dream and about his/her own participation in these events, but accepts them as real events, and it helps him/her to be totally involved in dream story, to participate in it with all vivid emotional feelings. For the dreamer it is not a mental game, it is a real life.

According to some authors (see Noreika et al., 2010) both lucid and non-lucid dreams are using a wide range of cognitive and meta-cognitive activities, and in non-lucid dreams speech and thought play an especially prominent role. From my point of view, they are used for the flexible SA without any rational control.

In wakefulness, even being totally involved in activity, subject is able to ask him-/herself: “What happens with me?” and to look on him-/herself and on his/her behavior as if it were from outside. However, it is necessary to emphasize that such estimation of the own behavior does not totally separate wakefulness from the non-lucid dreams. Even in dream, a feeling that something in subject's behavior is wrong and inappropriate may appear if it does not correspond to the feeling of “Self,” to the Self-Image (see Rotenberg, 2012) that is not lost in dream. But in contrast to the normal wakefulness, in dream it is only a general feeling, it is not a result of the analysis of the own behavior and of its possible outcomes that can change the direction of behavior. The Self-Concept, the ability to look at “Self” from outside is absent in non-lucid dreams and is present in lucid dreams, if a lucid dreamer understands what he/she is watching to is a virtual dream story. The peculiarity of the dream experiences in non-lucid dreams in comparison to the lucid dreams is related only to the absence of the self-reflection and of the estimation of him-/herself as a dreamer, of the self-person perspective that is present in wakefulness and in the lucid dreams (Kahn, 2007; Voss et al., 2009; Mota-Rolim et al., 2013).

Of course, the absence of self-person perspective and reflection in the typical non-lucid dreams has a great advantage for the main dream function which I have mentioned above. If such function is the restoration of SA as a process, it does not matter in what domain and in what direction it is oriented, subject's imaginations during dreams have to be very flexible and vivid, the content of dream can be immediately changed and subject has to be totally involved in this process with a doubtless feeling that it is his/her own real life. If a person has some doubts toward the reality of these experiences and is looking at them as if from outside, on what is going in dream, like in a cinema picture, he/she is losing a feeling of being inside in this story, and is no more excited by the events in the own dream and consequently, less active in his/her attempts. As a result, SA grew weak, because it is no more the subject's own life. It means that dream lucidity decreases the restorative potential of dreams and it is their disadvantage.

## The main task of lucid dreams: their advantage for search activity

So, does it mean that lucid dreaming is a failure of the dream functions? I suppose not. I think that lucid dreams have their own special function protecting subject from the cutting short the dream story when this story became traumatic for the subject. Lucid dreams allow dreamer to pass distressing and disturbing dreams that are functionally insufficient while being still sleeping, and may increase the renunciation of search instead of compensating it, thus to prevent terrible nightmares and to turn without awakenings step by step to the new dream stories that can perform their main function.

My explanation is very close to what La Berge has mentioned. According to La Berge, the insight that appears in lucid dreams and informs dreamer about what he/she is watching is nothing more than dream after some period passes, and lucid dream became displaced by the non-lucid dream. It is like a balance between the readiness to awake and to turn back to the normal sleep with the non-lucid dream (La Berge, 1985).

I believe that this supposition is confirmed by the nightmares treatment with lucid dreaming (Spooremaker and van den Bout, 2006). These authors have shown that in the process of such treatment, the number of nightmares' reports decreased. At the same time, there were no significant changes in subject's sleep quality and PTSD (post-traumatic stress disorder) symptom severity. Moreover, this treatment does not cause a stable reduction of nightmare frequency in general. It means that lucid dreams do not treat subjects from PTSD but can protect subject from the traumatic feelings of some concrete dream nightmares and allow subject to pass through it without awakenings. Thus, it is not a treatment of PTSD, it is a protection of sleep in the concrete dream. It is the reason of the reduction in dream recall (nightmare) frequency in subjects who succeeded in learning lucid dreaming (Holzinger et al., 2015)—they do not awake with a feeling of helplessness and it presents an opportunity to achieve the adaptive function of dreaming in the further dreams.

At this point, it is necessary to turn back to the essence of the functionally insufficient dreams that are not only common in patients with mental and psychosomatic disorders and are related to their pathogenesis, but may also episodically appear in healthy subjects. If the main function of dreams is the restoration of SA that was lost or decreased in different conditions during wakefulness, then the process of searching in dreams must be especially prominent after the renunciation of search in the stressful previous wakefulness.

Lucid dreams help to break the vicious circle between renunciation of search in wakefulness and sleep, because as soon as the dreamer realizes that he/she participates not in a real story but only in a dream, he/she became protected from the negative influence of this story on his/her own affect. Actually, it was shown that the so-called pre-lucid dreams that appear just before lucid dreams are often accompanied by strong negative emotions and feelings that subject who participates in the dream story is not protected from dangerous events. Such dreams may include, for instance, feeling of flying and fall (Hunt, 1989). In these conditions, to get an insight that it is only a dream is very supportive. It was also shown (Stumbrys et al., 2012, 2014) that in lucid dreams the dreamer is often able to influence the ongoing dream content. Lucid dreamers actively plan to accomplish different actions in their dreams like “talking” with dream characters, having sex and even flying (like it happens in non-lucid dreams), but sometimes, they are not able to successfully execute their intentions (like it happens in wakefulness) (Mota-Rolim et al., 2013).

Being in the lucid dream and looking on the dream story as if from outside, subject can continue to stay in REM sleep without awakenings. Because dream stories are in general very flexible, subject can after some period pass this trouble-making dream and turn to another dream that creates an opportunity for the SA. Lucid dream became displaced by the non-lucid dream and subject continues to sleep. Thus, the main advantage of lucid dreams is that these dreams protect the sleep process and help to pass the dreams that may cause nightmares and awakenings and to turn to the normal dreams that are restoring SA.

However, there may be also some other advantages. Bourke and Hannah (2014) have shown that subjects who have frequent lucid dreams are able to solve more insight problems in wakefulness in comparison to non-lucid dreamers. It corresponds to data (see Holzinger, 2009) that lucid dreams are reported by people with strong imagination, as well as to the investigation of Piller (2009) who showed the right hemisphere dominance during lucid dreams, because the right hemisphere plays an important role in creativity and insight (Rotenberg, 1993).

There are different views on the essence of lucid dreams in literature. According to above mentioned point of view, the lucidity is only the realization by the dreamer that the present experience is a dream, and all other features of the experience are similar in both types of dreams. Voss et al. (2009) explain lucid dreaming as a dissociative state combining cognitive elements of waking consciousness with the hallucinatory quality of dreaming. According to another point of view, lucidity may be sometimes accompanied by a full intellectual clarity, availability of the autobiographical memory sources and ability to control the dream content by the increase in the intensity of multimodal hallucinatory imagery (Metzinger, 2009). From my point of view, all these features of dream content are related to the main psychological characteristics of the lucidity—the ability to look on the dream from outside, as on the artificial imagined story created by the dreamer. As a story produced by the dreamer's mentality, it can be analyzed intellectually in details and it may be possible to find relationships between this story and the content of autobiographical memory. Of course, it is a difference between the pure lucid dreaming insight “it is only a dream!” that allows continue to sleep, and the intellectual dream control that makes the search in REM sleep dreams similar to the SA in wakefulness. In any case, whether dreams are lucid or not, they are separated from the information that is coming from the external environment that surrounds the dreamer.

If during wakefulness subject realizes his/her feelings and experiences as an own production, it can save subject from the uncontrollable outcome of these feelings. This represents conceptualization as a defense mechanism. Similar defense appears in lucid dreams—“it is my own dream, something not real.” It is a meta-cognition—the mental consideration of the own mental state and own virtual behavior, the realization of him-/herself as a cognitive subject. Meta-cognition is not totally absent also in non-lucid dreams, where what only is absent is a special form of meta-cognition that allows subject to recognize him-/herself being in a dream state, not in wakefulness. In non-lucid dream, subject can accept him-/herself as a person who is really doing something or perceiving something. It is the absence of the Self-Concept but self-feeling is not lost—a feeling that it is “I” who is doing it in these particular real conditions - and it determines the full involvement in the dream story—what is very important for the SA.

Non-lucid dreams represent primary consciousness similar to the consciousness before the development of Self-Concept as an outcome of reflection. The first-person perspective is less prominent in non-lucid dreams in comparison to wakefulness and often is absent, and the non-lucid dreamer is unable to form a conscious conceptual model of his/her current relations with the subjectively experienced dream world. Without the stable first-person perspective, dreamer not only is unable to realize that he/she is dreaming, but is also unable to direct voluntary attention and cognition at his/her own thoughts, emotions and behavior (see Noreika et al., 2010). From my point of view, the absence of the first person perspective and Self-Concept, the inability to feel him-/herself as an agent of the own behavior and the absence of the volitional and definitely directed self-control in dreams is a sign that the process of SA in dream is more important for the dream function than the conscious realization of him-/herself as a searching subject. For the same reason, subject often does not remember the content of dream soon after awakenings: this content has no sense by itself.

In lucid dreams, subject is separated from the virtual reality. It helps him/her to build a model of the own relationships with the dream world and to decrease emotional experiences caused by dreams, to became free from them at least for some period, but at the same time prevents the real awakenings. The realization that it is only a dream presumably does not block the chaotic jumps from one dream content to another, and finally, the dreamer can create a story that is less threatening and does not cause helplessness and turns to the non-lucid dream. It is the important advantage of lucid dreams.

## Conclusion

The task of this article was to elucidate the role of dream lucidity in the REM sleep dreams functions. According to the Search Activity Concept, this function is to restore the active behavior (search activity, SA) that dropped during the previous (pre-sleep) wakefulness, to increase it for the adaptation to the environmental demands in the post-sleep wakefulness. To achieve this goal, the content of dreams has to be vivid and challengeable and the subject's virtual behavior in dream must be active and flexible. The dream stories that were leading to failures have to be displaced by dream stories that stimulate search for problem solution.

If the renunciation of search (giving up and feeling helpless) dominates in dreams, like it happens in nightmares or in dreams of depressed patients, dreams are losing their adaptive function and frustrate the dreamer. Exactly in this condition, may help the dream lucidity. It is not the improvement of dream content, but it is an insight that what the subject is looking on is only a dream. A dreamer is looking in the lucid dream on him-/herself and on the dream story as if from outside. For this reason, he/she is not totally immersed by the traumatic dream content, as he/she is usually immersed in non-lucid dream. It is the disadvantage of lucid dream that does not allow lucid dream to perform the adaptive dream function. However, lucid dreams usually start when this adaptive function was already totally or partly lost, and in this condition, dream lucidity allows dreamer not to awoke in a state of frustration, but to pass the disturbing dream content, to continue to sleep and finally to turn to another dream that can be adaptive. It is the main advantage of lucid dreaming.

This hypothesis can be checked by the comparison of the number of lucid dreams (estimated by using volitional non-verbal signals presented by the dreamer during lucid dreams, see La Berge, 2000) with the reports presented after awakenings in non-lucid dreams, as well as with the number of spontaneous awakenings from REM sleep after strong emotional experiences.

### Conflict of interest statement

The author declares that the research was conducted in the absence of any commercial or financial relationships that could be construed as a potential conflict of interest.
